# Matters of Special Interest to the Association and to the General Profession of the State

**Published:** 1886-06

**Authors:** R. J. Nunn

**Affiliations:** President, Savannah, Ga.


THE ATLANTA MEDICAL AND SURGICAL JOURNAL.

VOL. III. JUNE, 1886. No. 4.

### Original Communications.

### MATTERS OF SPECIAL INTEREST TO THE ASSOCIATION AND TO THE GENERAL PROFESSION OF THE STATE.

AN INAUGURAL ADDRESS DELIVERED BEFORE THE MEDICAL ASSOCIATION OF GEORGIA AT AUGUSTA, 21ST APRIL, 1886.

BY R. J. NUNN, M. D., PRESIDENT, SAVANNAH, GA.

(*Concluded.*)

GENTLEMEN—I desire particularly to direct your attention to matters which concern the prosperity of the Medical Association of Georgia and the well-being of the profession in this State, to the end that such action may be taken as in your wisdom you shall determine may be best calculated to insure the welfare of both.

### LARGE MORTALITY AMONG THE MEMBERS.

I would particularly direct attention to the extraordinary death rate reported for the last year—no less than 11 out of a membership of 264, equal to 4.2 per cent. or 42 to the 1,000. The Committee on Necrology might with advantage investigate this subject.

THE LAW REGULATING THE PRACTICE OF MEDICINE.

This feature of our medical system invites your serious consideration. The law is as follows :

SEC. 1409 (a) *Who may Practice Medicine.* No person shall practice medicine within this State, unless he has been heretofore legally authorized so to do or shall be hereafter authorized so to do, by a diploma from an incorporated medical college, medical school or university and by compliance with subsequent sections of this chapter.

SEC. 1409. (b) *"Practice Medicine" Defined* for the Purposes of this Chapter. The words, "practice of medicine," shall mean to suggest, recommend, prescribe or direct for the use of any person, any drug, medicine, appliance, apparatus or other agency, whether material or not material, for the cure, relief or palliation of any ailment or disease of the mind or body, or for the cure or relief of any wound, fracture, or other bodily injury, or any deformity, after having received, or with the intent of receiving therefor, either directly or indirectly, any bonus, gift or compensation.

SEC. 1409. (c) *Practitioners must Register.* Every person now lawfully engaged in the practice of medicine within this State shall, on or before the first day of December, 1881, and every person hereafter duly qualified to practice medicine shall, before commencing the practice, register in the office of the clerk of the Superior Court of the county where he resides and is practicing or intends to commence the practice of medicine, in a book to be kept for that purpose by said clerk, his name, residence and place of birth, together with his authority for practicing medicine, as prescribed in this chapter. The person so registering shall subscribe, or verify by oath or affirmation, before a person duly qualified to administer oaths, under the laws of this State, an affidavit containing such facts, and whether such authority is by diploma or license, and the date of same, and by whom granted, which shall be exhibited to the county clerk before the applicant shall be allowed to register, and which if willfully false shall subject the affiant to conviction and punishment for false swearing. The county clerk to receive a fee of fifty cents for each registration, to be paid by the person so registering.

SEC. 1409. (d) *Must Register Again on Removal.* Any registered physician in this State, who may change his residence from one county into another county in this State, shall register within the clerk's office of the county to which he removes, and wherein he intends to reside and to practice medicine, as provided in the preceding section.

SEC. 1409. (e) *Penalty for Illegal Practice.* Any person who violates any of the four preceding sections, or who shall practice or offer to practice medicine without lawful authority, or under cover of a diploma or license illegally obtained, shall be deemed guilty of a misdemeanor, and on conviction shall be punished by a fine of not less than one hundred dollars or more than five hundred dollars, or by imprisonment for not less than thirty or more than ninety days, or both. The fine when collected shall be paid, the one-half to the person, persons or corporation making the complaint, the other half to the county treasurer.

SEC. 1409. (f) *Medical Officers Excepted.* Nothing in this chapter shall apply to the commissioned medical officers of the United States, army or navy, or to the United States marine hospital service, or to legally qualified dentists in the practice of their profession, or to any woman practicing only midwifery.

SEC. 1409. (g) *All Medical Boards Abolished.* All provisions of laws providing for the organization, qualification and duties of any and all boards of physicians of any school whatever are repealed. And there shall henceforth exist in this State no board of physicians, but the only requisite qualifications of practitioners of medicine shall be those hereinbefore set forth.

[The Code of the State of Georgia, 1882.]

I procured copies of the registration lists from some of the principal cities in Georgia. The aggregate number of names registered amounted to 433, purporting to be issued by 168 licensing powers of different names, although in many of them the same body is certainly referred to under varying titles.

The licensing bodies are distributed through twenty-six States and countries as follows: Alabama, Austria, Belgium, Canada, D. C., England, Georgia, Germany, Illinois, Iowa, Kentucky, Louisiana, Michigan, Massachusetts, Missouri, Maryland, New Hampshire, New York, Ohio, Pennsylvania, Prussia, South Carolina, Scotland, Syria, Tennessee, Virginia. And besides, there are a number whose location could not possibly be determined.

The following can hardly be considered as coming up to the very slender requirements, even of a Georgia law, regulating the practice of medicine:

(1) From Dr. Samuel Thompson, (2) Royal College of Dental Surgery, (3) Baltimore College of Dental Surgery, (4) Licentiate, (5) Charleston, (6) Philadelphia, (7) Atlanta, Georgia, (8) Licensed by Act of General Assembly. I also find one person whose sole registered authority to practice is an "*ad eundum*" degree, but what the original "*gradum*" was, is not stated.

None of these comply with the law, which uses the word medical before the words college or school.

In the following list the law is to all appearances complied with, in that the name apparently of an incorporated Medical College, Medical School or University, is given with one exception. Under which of these heads a State Board comes I cannot say. It will be observed that no state or city is mentioned by which the least clue to the location of these bodies could be obtained. Some of them we may know, or think we recognize, but that is not the question now. My object is to point out some of the radical defects in the law as it exists.

Here is the list:

(1) American College Medical and Surgeons, (2) College American Medical and Surgery, (3) Hygiene Therapeutic College, (4) American Reform School, (5) Jefferson College, (6) Jefferson Medical College, (7) Howard University, (8) Southern

Botanic Medical College, (9) Southern Eclectic Medical College, (10) Southern Medical College, (11) State Board Eclectic Physicians.

In the next class I will take up a series of names, in each of which the name of a city appears, to show the difficulty of identification when the name of the State is omitted.

*Atlanta*—(1) Southern Medical College of Atlanta, (2) Atlanta Eclectic Medical College, (3) Atlanta Medical College.

There are thirteen Atlantas—Arkansas, California, Georgia, Idaho, Illinois, Kansas, Louisiana, Michigan, Mississippi, Missouri, Nebraska, Ohio, Texas.

*Augusta*—Medical College, Augusta.

There are twenty Augustas—Arkansas, Dakota, Georgia, Illinois, Indiana, Iowa, Kansas, Kentucky, Maine, Michigan, Minnesota, Mississippi, Missouri, Montana, New Jersey, New York, Ohio, Texas, Virginia, Wisconsin.

*Baltimore*—College Physicians and Surgeons, Baltimore.

There are three Baltimores—Dakota, Maryland, Ohio.

*Boston*—Bellview Medical College, Boston.

There are twelve Bostons—Arkansas, Georgia, Indiana, Kentucky, Massachusetts, Missouri, New York, Ohio, Pennsylvania, Tennessee, Texas, Virginia.

*Charleston*—Charleston Medical College.

There are sixteen Charlestons—Arizona, Arkansas, Illinois, Iowa, Maine, Michigan, Mississippi, Missouri, New York, North Carolina, Pennsylvania, South Carolina, Tennessee, Texas, Utah, West Virginia.

*Chicago*—Medical College, Chicago.

There are six Chicagos—Illinois, Kansas, Kentucky, Nebraska, New York, Ohio.

*Cincinnati*—Medical Department of the Cincinnati College.

There are eight Cincinnatis—Arkansas, Illinois, Indiana, Iowa, Missouri, Nebraska, Ohio, Texas.

*Edinburg*—University of Edinburg.

There are nine Edinburgs—Dakota, Illinois, Indiana, Mississippi, Missouri, New Jersey, New York, Ohio, Pennsylvania

*Louisville*—(1) Louisville Medical College, (2) Louisville College of Medicine.

There are fourteen Louisvilles—Alabama, Colorado, Georgia,

Illinois, Kansas, Kentucky, Maryland, Mississippi, Missouri, Nebraska, New York, Ohio, Tennessee, Wisconsin.

*Macon*—(1) Botannic Medical College, Macon; (2) College of American Medicine and Surgery, Macon; (3) Reform Medical College, Macon; (4) Southern Botannic Medical College, Macon.

There was once, I believe, one medical college in Macon, Ga., but which one of these names refers to it I certainly cannot say, but to find out the location of the other three colleges will be a matter of some difficulty, for there are ten (10) Macons in as many different States, viz.: Arkansas, Florida, Georgia, Illinois, Michigan, Mississippi, Nebraska, North Carolina, Tennessee, Virginia. To which of these does the name in the affidavit apply?

*Nashville*—Nashville Medical College.

There are thirteen Nashvilles--Arkansas, California, Dakota, Georgia, Illinois, Indiana, Iowa, Michigan, Missouri, New York, North Carolina, Ohio, Tennessee.

*New Orleans*—New Orleans School of Medicine.

This one is not difficult to locate, there is but one New Orleans.

*Philadelphia*—University of Medicine and Surgery, Philadelphia, University of Philadelphia, Medical College, Philadelphia, Philadelphia Medical College.

There are eight Philadelphias—Illinois, Indiana, Mississippi, Missouri, New York, Pennsylvania, South Carolina, Tennessee.

*Savannah*—Savannah Medical College, Medical College of Savannah, Oglethorpe College of Savannah.

There are nine Savannahs—California, Georgia, Iowa, Missouri, New York, North Carolina, Ohio, Tennessee, Virginia.

*St. Louis*—American Medical College, St. Louis; Jefferson Medical College, St. Louis.

There are six St. Louis'—Arkansas, California, Indiana, Michigan, Missouri, Montana.

The following is a list of colleges registered, classified by States or countries. It will be observed that in many instances there is no guide, so that the classification is only a geographical surmise at best. Some, which could not by any possibility be located have been already mentioned.

*Alabama*—Alabama Medical College.
*Austria*—(1) University of Vienna, Austria; (2) University of Austria.
*Belgium*—(1) University of Brussels.

*Canada*—(1) College of Physicians and Surgeons, Ontario, Canada; (2) Victoria University, Upper Canada.

*District of Columbia*—(1) Columbia Medical College, D. C.; (2) Georgetown College, D. C.; (3) Howard University.

*England*—(1) Royal College of Physicians, London.

*Georgia*—(1) Atlanta, Ga., (2) Atlanta Medical College, (3) Southern Medical College of Atlanta, (4) Atlanta Eclectic Medical College; (5) Medical College, Augusta; (6) Medical College, Augusta, Ga.; (7) University of Georgia, Augusta; (8) Georgia Medical College, Augusta; (9) Medical College of Georgia, Augusta, Georgia; (10) Botanic Medical College, Macon; (11) College of American Medicine and Surgery, Macon; (12) Reform Medical College, Macon; (13) Reform Medical College, Macon Georgia; (14) Southern Botanic Medical College, Macon; (15) Medical College, Savannah; (16) Oglethorpe College, Savannah; (17) Oglethorpe Medical College, Savannah; (18) Savannah Medical College; (19) Board of Examiners of Georgia, (10) Board of Physicians of Georgia, (21) Botanic Medical Board of Georgia, (22) Georgia Eclectic Board, (23) Georgia Eclectic Medical College, (24) Georgia Medical Board, (25) Georgia Medical College, (26) Medical College of Georgia, (27) Medical Department, University of Georgia, (28) Reform Medical College of Georgia, (29) Southern Medical College of Georgia.

*Germany*—(1) Jenna Medical College, Germany, (2) Medical College at Giessen, (3) Strassburg University (Ger.), (4) University of Liepsic.

*Illinois*—(1) Bennett Medical College, Chicago; (2) Russ Medical College, Chicago; (3) Rush, of Chicago.

*Iowa*—(1) College of Physicians and Surgeons, Keokuk, Iowa.

*Kentucky*—(1) Hospital Medical College, Louisville, Ky.; (2) Kentucky School of Medicine, Louisville; (3) University of Louisville, Ky.; (4) Hospital College of Medicine, Louisville; (5) Kentucky School of Medicine; (6) Medical Department Central University, Kentucky; (7) Transylvania Medical College, Ky.; (8) University of Kentucky.

*Louisiana*—(1) New Orleans School of Medicine, (2) New Orleans School of Medicine, La.; (3) University of Louisiana.

*Maryland*—(1) Baltimore College of Dental Surgery; (3) College of Physicians and Surgeons, Baltimore, Md.; (4) Medical Department University, Md.; (5) University of Maryland, (6) University of Maryland, Baltimore; (7) Washington University, Baltimore.

*Massachusetts*—Worcester Medical College, Mass.

*Michigan*—(1) College of Medicine and Surgery, Ann Arbor, Michigan; (2) University of Michigan.

*Missouri*—(1) Hahnemann Medical College, St. Louis.

*New Hampshire*—(1) New Hampshire Medical College.

*New York*—(1) Bellevue Medical College, New York; (2) Bellevue, New York; (3) College of Physicians and Surgeons, New York city; (4) Eclectic Medical College, New York; (5) Homœopathic Medical College, New York; (6) Long Island College and Hospital, New York; (7) Medical Department University of New York; (8) Metropolitan College, New York; (9) New York Homœopathic College; (10) New York Hygiene Therapeutic College; (11) Physicians and Surgeons, New York; (12) University, city of New York; (13) University of New York; (14) Bellevue Hospital Medical College.

*Ohio*—(1) Cleveland Homœopathic College, Ohio; (2) Eclectic College, Cincinnati, Ohio; (3) Eclectic Medical College, Cincinnati, Ohio; (4) Homœopathic College, Cleveland, Ohio; (5) Miami Medical College, Cincinnati, Ohio; (6) Ohio Medical College, Cleveland; (7) Starling Medical College, Columbus, Ohio; (8) Cincinnati Eclectic Medical College; (9) Medical Department Cincinnati College; (10) American Eclectic College of Ohio; (11) American Health College, Ohio; (12) The Physic Eclectic College of Ohio.

*Pennsylvania*—(1) Jefferson Medical College of Philadelphia, Penn; (2) Hahnemann Medical College of Philadelphia, Penn.; (3) Homœopathic Medical College of Pennsylvania, (of Philadelphia.); (4) Medical Department of the University of Pennsylvania, Philadelphia; (5) University of Pennsylvania, of Philadelphia; (6) American University, Philadelphia; (7) Eclectic Medical College, Philadelphia; (8) Hahnemann Medical College, Philadelphia; (9) Jefferson Medical College, Philadelphia; (10) Eclectic Medical College of Pennsylvania; (11) Jefferson Medical College, Pennsylvania; (12) Medical Department Pennsylvania College; (13) Medical College, Pennsylvania, (14) Pennsylvania Eclectic Medical College; (15) Pennsylvania Electropathic Institute; (16) Pennsylvania Medical College; (17) University of Pennsylvania; (18) Woman's Medical College of Pennsylvania, Philadelphia.

*Prussia*—(1) Royal University, Berlin.
*South Carolina*—(1) South Carolina Medical College of Charleston, South Carolina; (2) Charleston, (3) Medical College of South Carolina, (4) South Carolina Medical College.
*Scotland*—(1) University of Glasgow, Scotland.
*Syria*—(1) The Syria Protestant Medical College at Brent.
*Tennessee*—(1) College of Central Tennessee, Nashville; (2) Medical College, Nashville, Tennessee; (3) Nashville, Tennessee (4) The Old University of Nashville, Tennessee; (5) University of Nashville, Tennessee; (6) University of Nashville, (7) Vanderbilt University, Nashville; (8) Medical Department of Central College, Tennessee; (9) University of Tennessee, (10) Vanderbilt University of Tennessee.
*Virginia*—(1) Medical College of Virginia, (2) University of Virginia, (3) Virginia.

Fully 3 per cent. have registered qualifications unauthorized by the law, which is rather singular, as in cities where competition is greatest we would naturally expect to find the law strictly enforced, and if such looseness is manifested there, the number of disqualified practitioners still practicing medicine mustbe very great.

If this subject is considered by the Association to be worthy of investigation, a share of its attention might also well be devoted to the pernicious, dangerous and illegal practice of counter-prescribing by druggists and druggists' clerks now prevailing in towns to an enormous extent.

In one city, with a roll of forty (40) physicians in the directory, I found ten (10) unregistered. If this proportion is true, of the rest of the State there are five hundred (500) physicians who have not complied with the provision of the law.

It has been stated that in the city of Berlin, Germany, there are 3,400 persons holding diplomas purchased in this country; surely there is no reason to suppose that the same class does not exist in Georgia, but our law requires no proof of the genuineness of the document registered, nor of steps taken to procure it, whether by purchase or by study and examination. Here one of Dr. Buchanan's ten dollar diplomas is just as legal as that of the best college in the world acquired after years of study.*

---

"By an edict of the Berlin State Court," says a writer of German notes in the Chicago *Tribune*, "the diplomas of M. D. bought of a now defunct medical university of Philadelphia, the holders of which in Germany were humorously styled 'Doctores Philadelphiæ,' are declared invalid, and the prosecution of such persons who persist in advertising themselves as 'specialists' in Germany on the strength solely of such diplomas is authorized. The happy possessor of such a diploma, a 'Dr.' Reseck, in Berlin, was fined 300 marks for calling himself a physician, and 150 marks for wearing the title of 'M. D.'"

The law, as now interpreted and applied, has proved to be practically useless in that—

1. It permits any person possessing or professing to possess in his own name a diploma from an incorporated medical college, medical school or university, to practice medicine.
2. It provides no test of the value of the diploma so that one of the Buchanan type is as good as any other.
3. The licensee is not required to exhibit his diploma, to the registrar, nor any evidence that he ever had one, except his own unsupported affidavit.
4. It is not made the duty of any official to inquire whether the affidavits filed with the county clerk, under provisions of the Act, are false or true.
5. It is not made the duty of any one to see that the provisions of the Act are carried out—consequently they are neglected.
6. The law does not provide for giving the post-office address of the institution granting the diploma, so that it is impossible to test the authenticity of the document.
7. That 25 per cent. of the physicians of one city should be still unregistered shows that the law is inoperative.
8. That names other than the corporate names of colleges are sworn to by the licensees shows on their part a very great disregard of the law, and of the value of an affidavit.
9. That persons are practicing upon qualifications not authorized by law demonstrates either that the law is defective or the officials negligent.

It is not strange that our officials, in obedience to the clamor of partisans should be active in hunting down gamblers, liquor sellers and Sunday law-breakers, nor is it at all extraordinary that laws little cared for by the people should be neglected. But it certainly is a curious phase in the character of the educated portions of a community, which encourages the success of ignorance over education, and passively submits to the evasion of every law made, especially for the protection of the feeble, the helpless and the ignorant. Stranger it is to see men pretending to some refinement and education struggling vigorously in time of health to lower the standard of education of the very men from whom in emergency they demand the broadest information, and upon whose knowledge and skill they depend when sickness, pestilence and death stare them in the face; but stranger yet than all is the sight of a legislature granting a license to practice to a person whose sole qualification must have been his inability to pass a satisfactory examination before any of the bodies authorized to issue diplomas. "*Quem deus vult perdere prius dementat.*"

It is not impossible, however, that the ineffectiveness of the law is rather a fault of the careless interpretation placed upon it than the fault of the Act itself.

The Act of the State of Georgia is evidently modeled after that of the State of New York, of 1880, certain sections being omitted, among which is section 4.

The omission of section 4 either leaves those practitioners moving into the State, subject to the control of the incorporated medical colleges, medical schools or universities of the State, and compels them to submit to examination before these bodies, or else it permits the free influx of every medical mountebank, who may desire to reside within the hospitable borders of this paradise of quacks.

The latter interpretation is the one now given to the law, but that the Association may act advisedly in the matter, I append a copy of the law of the State of New York:

"AN ACT"

"*To Regulate the Licensing of Physicians and Surgeons—Passed May* 29, 1880.

"*The People of the State of New York, Represented in Senate and Assembly, do enac as follows*:"

"SECTION 1. A person shall not practice physic or surgery within the State, unless he is twenty-one years of age, and either has been heretofore authorized so to do' pursuant to the laws in force at the time of his authorization, or is hereafter authorized so to do, as prescribed by chapter seven hundred and fifty-six of the laws of eighteen hundred and seventy-two, or by subsequent sections of this Act.

"SEC. 2. Every person now lawfully engaged in the practice of physic and surgery within the State shall, on or before the first day of October, eighteen hundred and eighty, and every person hereafter duly authorized to practice physic and surgery, shall, before commencing to practice, register in the clerk's office of the county where he is practicing or intends to commence the practice of physic and surgery, in a book to be kept by said clerk, his name, residence and place of birth, together with his authority for so practicing physic and surgery as prescribed in this Act. The person so registering shall subscribe and verify by oath or affirmation, before a person duly qualified to administer oaths under the laws of the State, an affidavit containing such facts, and whether such authority is by diploma or license and the date of the same, and by whom granted, which, if willfully false, shall subject the affiant to conviction and punishment for perjury. The county clerk to receive a fee of twenty-five cents for each registration, to be paid by the person so registering.

"SEC. 3. A person who violates either of the two preceding sections of this Act, or who shall practice physic or surgery under cover of a diploma illegally obtained, shall be deemed to be guilty of a misdemeanor, and on conviction shall be punished by a fine of not less than fifty dollars nor more than two hundred dollars for the first offense, and for each subsequent offense by a fine of not less than one hundred dollars nor more thau five hundred dollars, or by imprisonment for not less than thirty days nor more than ninety days, or both. The fine when collected shall be paid, the one half to the person or corporation making the complant, the other half into the county treasury.

"SEC. 4 A person coming to the State from without the State may be licensed to practice physic or surgery, or either, within the State in the following manner: If he has a diploma conferring upon him the degree of doctor of medicine, issued by an incorporated university, medical college or medical school without the State, he shall exhibit the same to the faculty of some incorporated medical college or medical school of this State, with satisfactory evidence of his good moral character, and such other evidence, if any, of his qualifications as a physician or surgeon as said faculty may require. If his diploma and qualifications, then they shall endorse said diploma, which shall make it for the purpose of his license to practice medicine and surgery within this State the same as if issued by them. The applicant shall pay to the dean of said faculty the sum of twenty dollars for such examination and indorsement. This indorsed diploma shall authorize him to practice physic and surgery within this State upon his complying with the provisions of section two of this Act.

"SEC. 5 The degree of doctor of medicine lawfully conferred by any incorporated medical college or university in this State shall be a license to practice physic and surgery within the State after the person to whom it is granted shall have complied with section two of this Act.

"SEC. 6. Nothing in this Act shall apply to commissioned medical officers of the United States army or navy, or of the United States Marine Hospital service. Nor shall it apply to any person who has practiced medicine and surgery for ten years last past, and who is now pursuing the study of medicine and surgery in any legally incorporated medical college within this State and who shall graduate from and receive a diploma within two years from the passage of this Act.

"SEC. 7. All Acts or parts of Acts inconsistent with the provisions of this Act are hereby repealed."

As instances of what may be done toward the legitimate regulation of the practice of medicine, I would direct your attention to the States of Alabama, Illinois and Virginia, in which this Association may study with advantage the operations of a good medical law vigorously, faithfully and justly enforced. It will then be demonstrated that good medical laws are not obnoxious to a republican form of government.

Speed the day when Georgia will lift the black cloud from her medical profession.

It is remarkable that legislatures seldom rise to that noble philanthropy which would be expected of bodies of men entrusted with the power of making laws for the public good, but on the contrary they are not unfrequently themselves actuated by motives of fanaticism and narrow bigotry. These "men of strong opinions and weak parts,"* "whose minds are like the pupil of the eye, the more the light shines upon them the more they contract"†, will carry with them into the legislative halls their personal prejudices upon all subjects, not even excepting theology and medicine.

I regret exceedingly that the very late date on which I received the lists of registrations prevented a more thorough analysis of the operation of the law regulating the practice of medicine in the State of Georgia. I trust, however, that I have succeeded in presenting facts enough to demonstrate that the subject should receive some consideration from this Accociation.

If we cannot do better, let us endeavor to have the present law

* Toombs. † Holmes.

stricken from the statute books of the State. Better to boldly acknowledge that we have no State enactment regulating medical practice than that we should continue to deceive ourselves and the world with the mockery of a law now foisted upon us.

So much for the Code of 1882.

### ACT OF 1883.

### PARTURIUNT MONTES, NASCETUR RIDICULUS MUS.

The following is copied from "Acts and Resolutions of the General Assembly of the State of Georgia, 1882–83, compiled and published by authority." Atlanta, Ga., JAS. P. HARRISON, State Printer, 1883. Page 68, part I, title IV., Code amendments.

### REGULATING PRACTICE OF MEDICINE, NO. 413.

An Act to amend section 1409 (a) of the Code of 1882 in relation to who shall practice medicine by adding the following words: "or has, after attending one or more terms at a regular medical college, or were by law authorized to practice medicine and have been in active practice of medicine since 1866:"

SEC. I. *Be it enacted by the General Assembly of the State of Georgia, and it is hereby enacted by the authority of the same,* That from and after the passage of this Act, section 1409 (a) of the Code of 1882 be amended by adding the words "or has after attending one or more full terms at a regular chartered medical college, been in active practice of medicine since the year 1866," between the words "university" and "and" in the fourth line of said section, so that said section when amended shall read as follows: No person shall practice medicine within this State unless he has been heretofore legally authorized so to do, or shall be hereafter authorized so to do, by a diploma from an incorporated medical school or university, or has, after attending one or more full terms at a regularly chartered medical college, beenin active practice of medicine since the year 1866, or who were by law authorized to practice medicine in 1866.

SEC. II. *Be it further enacted,* That all laws and parts of laws in conflict with this Act be, and the same are hereby repealed.

Approved September 27, 1883.

A Western judge is said to have delivered a negative opinion in the following terms: "If the court understand herself, and it think she do, it aren't." It would be difficult to concentrate a greater number of mistakes into so small a compass, but the General Assembly of Georgia is deserving of credit for the effort made while tediously and painfully bringing forth this deformed Act to emulate the performance of the Western judge.

Even a Georgia Legislature has excelled itself in its efforts to if possible still further darken its already black record on the subject of medical legislation. By enacting a new law intended to pull down the door already left widely ajar by the Code, and furnish all comers, whether they have the least qualification or not, to practice medicine, which invites every medical showman with fife and drum, with monkey and hand-organ, with a band of negro minstrels, with pantomime dress or with any of the thousand claptrap appliances adopted by the itinerant fraternity, to visit our State and swindle our people if by good chance their criminality extends no farther.

It is difficult to decide which is most to be admired, the ingenuity of the framer of this bill or the courage of the Legislature that passed it—evidently without proper consideration. But between them they have added another laurel to the brow already notorious among the educated and thinking classes for enacting laws to foster medical ignorance and to encourage and protect dishonest debtors. Will the sentiments of the honorable gentlemen who composed the good old *ante-bellum* legislatures never again find voice in Georgia?

| 1. | 2. | 3. | 4. |
|---|---|---|---|
| *Section* 1409 (*a*) *Code*, 1882.—No person shall praciice medicine within this State, unless he has been heretofore legally authorized so to do, or shall be hereafter authorized so to do, by a diploma from an incorporated Medical College, Medical School or University. | *Amended as Per Caption of bill of* 1883.— | *Amended as per body of bill of* 1883.— | *The Amended Section as recited in bill of* 1883. |
| | or has, after attending one or more full terms at a reguiar ——— medical college, or who were by law authorized to practice medicine, and have been in active practice of medicine since 1866. | or has, after attending one or more full terms at a regular chartered medical college | or has, after attending one or more full terms at a regularly chartered medical college |
| | | been in active practice of medicine since the year 1866. | been in active practice of medicine since the year 1886, or who were by law authorized to practice medicine in 1866. |
| and by compliance with subsequent sections of this chapter. | | | |

For convenience of reference I have placed in juxtaposition, 1st, section 1409 (a) Code of 1882; 2nd, the same as amended in accordance with the caption of the Act of 1883; 3rd, the same as amended according to the body of the Act of 1883–4, the amended section as given in the Act of 1883.

A careful comparison of these four forms will develop some extraordinary facts: 1st. Different terms are used to qualify the word "college" in 2, 3, 4, so that it becomes a matter of impossibility to determine what it is intended to convey.

This crude bill bears the stamp of illiteracy so strongly impressed upon it that there can be no hesitation in pronouncing it the handiwork of one of that ignorant class which the operations of the "higher law" has liberally bespattered and besmirched our Legislature.

In the caption of the bill, the words "REGULAR *medical college*" are used, in the body of the bill the words "REGULAR CHARTERED *medical college*" are employed, while in the so-called *amended* section the words "REGULARLY CHARTERED *medical college*" are introduced.

When dealing with technical subjects one should, if using technical language, use it correctly, or else the terms employed should be defined. Did the framer of this Act mean a *regular* medical college, as distinguished from an *irregular* medical college? What is meant by "*regular chartered medical college*"? All *regular* medical colleges have charters. What is to be understood by the phrase, "REGULARLY *chartered medical college*"? The notorious colleges of Philadelphia, and others of the same class, are or were regularly chartered.

Already, perhaps, too much time has been occupied with this phrase, and I will now direct your attention to another.

2. The words "or who were by law authorized to practice medicine" occurring in No. 2 (caption) are omitted in 3 and are transposed in No. 4 so that what is made obligatory in No. 2 becomes an optional alternative in No. 4.

3. The words "and have" occurring in No. 2 are entirely omitted in Nos. 3 and 4.

4. The words "and by compliance with subsequent sections of this chapter" occurring in No. 1 (Code, 1882) and not referred to in No. 2 (caption of 1883) are entirely omitted in No. 4: this omission taken in connection with the repealing section (section 2) of this bill, actually repeals this provision of the Code of 1882.

5. The words "active practice" used in 2, 3 and 4 need explanation. The Code of 1882 explains what "the words practice of medicine shall mean," but the qualifying word "active" implies more than one kind of practice, such, for example, as its opposite "*passive*." These varieties of practice are not known to physicians.

6. The sentence "been in active practice since 1866" is ambiguous. It may mean "been in active practice" at any time "since 1866, or it might be construed to mean "been in active practice" continually "since 1866." I know that both constructions have been claimed for it.

7. The Act does not specify in what part of the world the practioner is to have had his experience in active practice, so that it is not impossible that by placing a liberal interpretation upon the language of the Act an Indian medicine-man or an African obee or voodoo would be hailed with delight by the General Assembly of Georgia, and marshaled before the world as belonging to the medical profession of this State and educated in accordance with advanced and advancing ideas of a Georgia Legislature.

8. The bill, while repealing the registration provision of the Code of 1882, fails to substitute any equivalent test.

9. The bill does not give authority to any one nor does it under the Act become the duty of any one to inquire into facts professedly, ostentatiously and deceptively required by the language of the Act.

10. There is absolute silence in the bill touching what shall constitute evidence of the *high* qualifications required by the bill.

11. The phrase "after attending one or more full terms at a regular medical college," or "a regular chartered medical college," or "a regularly chartered medical college," is misleading, because, while personally present the student might be reading a newspaper or be otherwise preoccupied, and might not hear a word said by the lecturer, or hearing the words might not understand their meaning.

12. If it is intended by this bill to require evidence that an individual has attended every lecture of a term, then the bill requires an impossibility. There is not a registry of the attendance kept in any college in the United States, if there is in the world. The possession of lecture tickets is no evidence of attendance, as the tickets are bought before the lectures commence, or at least are so supposed to be.

13. A trifling matter, but one showing the illiteracy of the framer of this bill, is the use of a verb in the singular and a verb in the plural, both referring to the same noun in the singular, "No person shall . . . unless . . . . , or *has* . . . or *who were*."

If I, who am not a lawyer, and have not the least legal acumen, can discover so many mistakes, inaccuracies, and inconsistencies in an Act of twenty-two lines, how many would be found by an astute and competent lawyer? And now a neat and interesting little arithmetical problem suggests itself: If twenty-two (22) lines of Georgia law contains not less than thirteen (13) errors, how many occur in the whole volume?

In the pedantic language of the classical center of the country, this bill would be called "a perditioned Machiavelian machination."

It is notorious that illiteracy and ignorance are in the ascendency in the houses of the State Legislature, but the Chief Executive has never been either illiterate or ignorant, but that laws of such a character as the one under consideration should have received his sanction, plainly shows that there is an utter want of attention to business, a neglect of things which have the appearance of being small; evidently the stamp of approval has been placed upon this law without any knowledge of the import of the bill. The responsibility for this gross negligence of duty rests somewhere, and should be placed where it belongs.

There are sins of omission and of commission, of ignorance, of negligence and of intention. How the baker's dozen of errors in this twenty-two line bill are to be classified and how divided between the parties guilty of giving vitality to this Act, must be left to the parties themselves to determine, with a perfect confidence, however, that they will not quarrel with each other for the largest share of the honors.

Had this been a matter of a few dollars the august Legislature would have critically examined every line of the Act and spent more of the people's money in vacuous speeches than the amount under discussion, as was done in the case of the State University at the last session ; but this little bill of 1883 was only a question of education ; it only affects the life and health of the people; it does not apparently touch the public pocket, and therefore these men, sworn to look after the public welfare, think they can conscientiously neglect their duty. If, on the other hand, they discussed at great length the paltry appropriation asked for the State University, it must not be forgotten that this course enabled these solons to pose before their constituents as public economists of the first water, forgetting to mention, however, that while apparently saving a few hundred dollars for the public treasury, they talked double the sum out of the State exchequer and into their own pockets.

Under a monarchical government,such as England, when classes of business or professions are likely to be affected by legislation, representative men from those opposed and from those in favor of the proposed changes are examined before a committee and the law-making power acts prudently and cautiously, while in the great commonwealth of Georgia, the Legislature, instead of consulting the wishes and welfare of a large body of citizens, permits the ideas of an individual to be smuggled into a law. This certainly is just the opposite of what might have reasonably been expected under this different form of government.

When about to legislate upon technical matters, of which of necessity the Legislature must be profoundly ignorant, common prudence, that prudence which each individual would exercise in his own business, would dictate that counsel would be taken of experts, of men who are in front rank of progress in that particular branch of science; and if such are not to be found within the limits of the State, or if those within the State are not trustworthy, they can be found outside. Thus, in questions of medical education, the legislation of Alabama, of Virginia, of Illinois, of England, France or Germany, might be consulted, but certainly the sweet will of the ignorant and illiterate member from Tarbucket county, who signs his name with a X, should not be allowed to influence the action of the Legislature even though he desired to benefit and serve some illegitimate pill-peddler equally as ignorant and illiterate as himself.

Each Legislature, instead of devoting itself to wise and permanent legislation, seems rather to be intent upon finding some way in which to change the Acts passed by its predecessors, and passing such Acts as its successor will easily find cause to change, like some prudent mechanics who leave "a screw loose" so as to secure another job in the near future, either for himself or somebody else.

There are some educated and refined gentlemen in the General Assembly of Georgia, and certainly the tax-payers have a right to demand that these gentlemen will see that the laws are prepared in intelligible English, despite the liberties permitted in discussion, and that when a phrase is repeated it is not changed; that transpositions of sentences, with consequent changes of meaning, are not permitted unless it is in accordance with the caption of the bill, and that liberties are not taken with existing laws which are not in accordance with caption of the bill making the change.

It is a serious question if this bill was not obtained to be smuggled through the Legislature by some of the quack itinerants who are now—thanks to the combined efforts of our enlightened General Assembly and of our careful Chief Executive—favoring the people of Georgia with exhibitions of their wonderful healing powers, emulating in this particular even Divinity itself.

Driven from other States by wholesome medical laws, and unwilling or unable to enter the profession of medicine, through the door of education, they naturally gravitate to Georgia, the Empire State of the South, whose *imperial* law-givers, actuated by a spirit of exalted wisdom, high cultivation and advanced civilization, have chosen to put medical ignorance at a premium and medical education at a discount.

It was one of this fraternity who, when called upon to register under the Code of 1882, by the proper officer, refused to do so, and directed the attention of the official to the *scrub law* of 1883.

I could pursue this analysis to much greater lengths, but I think what has been said is sufficient to show how the public servants of this State are performing their duty, and it may be that the educated and enlightened element in the State may take this condition of things into serious consideration, and take steps to correct this serious evil.

Upon this subject see also *The Physicians' Magazine* for March, 1886, p. 220, et. seq.

THE ACT PROPOSED BY THE AMERICAN MEDICAL ASSOCIATION.

The American Medical Association at its last meeting prepared and ordered to be sent to the various State associations, for their action, an Act entitled "An Act to Establish a State Board of Medical Examiners and Licensers, and to Define the Duties and Powers of such Board:"

SECTION I. *Be it enacted by the Senate and House of Representatives of the Commonwealth of ——— in General Assembly met, and it is hereby enacted by the authority of the same*, That there shall be appointed by the Governor a State Board of Medical Examiners and Licensers, consisting of nine members, three of whom shall serve for one year, three for two years and three for three years; and hereafter he shall each year appoint three members to serve for three years, in place of those whose terms then expire. They shall be graduates of some legally chartered college or university, having the power to confer medical degrees, who shall have practiced medicine or surgery for a period of not less than five years, but none of whom shall be members of the faculty or of any such college or university; *Provided*, that in the appointment of said board, at least seven members shall be chosen from a list of twenty-one names, submitted by the medical society of the State of ———. In default of the submission of such list the Governor shall appoint ——.

SEC. II. Upon the organization of said board, it shall be determined by lot which three members shall serve for a term of one year, which three for a term of two years, and which three for a term of three years Every appointment to fill a vacancy or vacancies in the said State Board of Medical Examiners and Licensers shall be for the unexpired term, and the said vacancy or vacancies shall be filled by the Governor within sixty days after notice to him of the same; *Provided*, that when the vacancy has been caused by death, resignation or removal of a member appointed from the list furnished by the medical society of the State of ———, the said vacancy shall then be filled from a list of three names for each vacancy furnished by the medical society of the State of ———. In default of the submission of such list the Governor shall appoint———.

SEC. III. The said board shall be a corporation by the name and style of the State Board of Medical Examiners and Licensers of the Commonwealth of ——, and shall have and use a common seal, and as such corporation may sue and be sued, contract and be contracted with, plead and be impleaded to the extent to enable it to carry out the powers conferred upon it by this Act. Said board may make and adopt all necessary rules, regulations and by-laws, not inconsistent with the constitution and laws of this commonwealth, or of the United States, to enable it to perform its duties and transact its buisness under the provisions of this Act; and shall, upon its organization, elect from its own number a President and a Secretary, who shall also act as Treasurer, both of whom shall hold their offices for a term of two years.

SEC. IV. Every person who shall be appointed to serve on said State Board of Medical Examiners and Licensers, in the manner aforesaid, shall receive a certificate of appointment from the Governor, and within thirty days after receiving a certificate of his appointment, shall file the same with the prothonotary of the Court of Common Pleas in the county in which he or she shall have previously registered under the present Acts of assembly, and shall also file a certificate of his or her said appointment as a member of said State Board of Medical Examiners and Licensers in the office of the Secretary of the State of the commonwealth of ———.

SEC. V. The said State Board of Examiners and Licensers shall examine all applicants for license to practice medicine or surgery in this commonwealth who are properly qualified, according to the provisions of section vii. of this Act, and shall exclude no one from examination, nor reject him or her because of his or her adhesion to a special system of practice. It shall hold two stated meetings each year, one at —— on the second Tuesday in May, and at —— on the second Tuesday in November, respectively, and may hold special meetings at such times as it may deem proper. All examinations shall be conducted in *writing*, and all examination papers, together with the reports and action of the examiners thereon, shall be preserved as the records of the said board for a period of five years, during which time they shall remain open for inspection at the office of the State Board of Medical Examiners and Licensers, which office shall be in ——.

SEC. VI. Such examination shall be in anatomy, physiology, general chemistry, pathology, therapeutics, principle and practice of medicine, surgery and obstetrics; *Provided*, that each applicant, upon receiving from the Secretary of the board an order for examination, shall also receive a confidential number, which he or she shall place upon his or her examination papers, so that when said papers are passed upon by the examiner, the latter shall not know by what applicant said papers have been prepared; that upon each day of examination all candidates be given the same set or sets of questions. It is further provided that the examination papers shall be marked upon a scale of 100, and that, in order to secure a license, it shall be necessary for the applicant to attain such average as shall hereafter be determined by the said State Board of Examiners and Licensers.

SEC. VII. Any person, on paying twenty dollars to the Secretary of said board, and on presenting satisfactory proof of being over twenty-one years of age, of good moral character, and of having received a diploma from any legally chartered college or university having authority to confer degrees in medicine, shall be entitled to examination by the said board, and ,in case of failure at any such examination, shall have the privilege of a second examination withou tthe payment of any additional fee.

SEC. VIII. For the purpose of examining and licensing applicants, as well as the transaction of other business, five members shall constitute a quorum of said board, and when the President and Secretary of said board shall find that an applicant has attained the necessary examination average, they shall issue to him or her a license to practice medicine and surgery in the State of ———.

SEC. IX. After the first day of September, 1886, no person shall enter upon the practice of medicine or surgery in the State of ———, unless he or she has complied with the provisions of this Act, and shall have exhibited to the prothonotary of the Court of Common Pleas of the county in which he or she resides, a license duly granted to him or her by the said State Board of Examiners and Licensers, upon which he or she shall be entitled, upon the payment of one dollar, to be duly registered in the office of the prothonotary of the Court of Common Pleas in said county, but nothing in this Act shall be so construed as to prevent the practice of medicine and surgery by any practitioner who shall have been duly registered before the first day of September, 1886, according to the terms of the present Act of assembly.

SEC. X. For the purpose of this Act the words "practice medicine or surgery" shall mean to treat or attend any person for money, gift or reward.

SEC. XI. Nothing in this Act shall apply to commissioned medical officers of the United States army or navy, or of the United States marine hospital service, nor to any member of the house or resident staff of any legally chartered medical college or university or hospital, during his term of service therein, nor to physicians of other States meeting duly registered physicians of this State in consultation, nor to those practicing dentistry exclusively, nor to midwives.

SEC. XII. The Secretary shall record, in a book to be kept for the purpose in the office of the said board, the name, age, sex, residence, date and place of graduation of each applicant, together with the date of the examination, the examination number, the examination average on each branch, the general average and date of issue of license, in case such license is granted. Said book shall be open to public inspection, and on or before the last day of December of each and every year, the said board shall publish, or cause to be published, a list of the names and addresses of such persons as shall have received licenses from the said board within twelve months immediately thereto preceding.

SEC. XIII. The members of the said board shall each receive a salary, not exceeding seven hundred dollars per annum, to be paid out of the fees for examination. The Secretary and Treasurer shall receive an additional salary, to be fixed by the board, and shall give bond, in the sum of one thousand dollars, that he or she will faithfully account for the sums paid into his or her hands. The balance of the fees, after the necessary expenses of the board, which must be stated by affidavit, have been deducted, shall be paid into the treasury of the commonwealth of——

SEC. XIV. The Governor may remove any member of the said board, for unprofessional or dishonorable conduct, upon the recommendation of a two-thirds vote of the said board.

SEC. XV. The sum of one thousand dollars is hereby appropriated to meet the necessary and legitimate expenses of the said board for the year commencing the first day of September, 1886.

SEC. XVI. This Act shall take effect on the first day of September, one thousand eight hundred and eighty-six.

SEC. XVII. Should any charge or charges of unprofessional conduct be preferred against any duly licensed practitioner of medicine or surgery, the said State Board of Medical Examiners and Licensers of the said commonwealth of———— shall have power to summon said practitioner before it, having previously given him or her fifteen days' notice of such charge or charges, with the name or names of the party or parties preferring the same, and the name or names of any witness or witnesses to be called and examined in support of said charge or charges, and upon hearing therefore, and having heard the party or parties so accused, and any witness or witnesses he or she may desire to call as to his or her defense, the said State Board of Medical Examiners and Licensers shall, upon satisfactory proof of the truth of said charge or charges, refer his or her case to the district attorney of the county wherein he or she shall have practiced, with all specifications and all evidence in support of the same, who shall apply for a rule upon the party so accused, to show cause why his or her license shall not be revoked, and the proper court shall have power so as to direct such licensee to be stricken from the list recorded in the office of the prothonotary of the court of common pleas for the county in which the said accused shall reside.

SEC. XVIII. Any person violating the provisions of this Act shall be guilty of a misdemeanor, and upon conviction thereof in the Court of Quarter Sessions of the county where the offense shall have been committed shall pay a fine of not less than fifty nor more than five hundred dollars for each offense.

I would suggest that it could be improved at least as follows:

1. Make the grade of examinations uniform in each State.
2. Provide for a system of representation between States.
3. Grant to graduates equal privileges in all States represented on the examining board.
6. Judges to give the matter in charge to grand juries.
7. Examinations should be oral and clinical as well as written.
8. The annual list should contain not only the names of those licensed during the year, but also the names of all persons licensed to practice medicine in the State.
9. The omission of a name from the directory should be *prima facie* evidence of disqualification to practice. (See English system).
10. It should be made the duty of some official to carry this law into effect.
11. Conflicting laws to be repealed.

### REPRESENTATIVES TO AND FROM OTHER STATE ASSOCIATIONS.

Sec. 3, article 5, of the constitution says of the president, "he shall appoint annually the requisite number of delegates to the American Medical Association and to such other scientific bodies as it may be expedient to have the association represented in."

It is very evident that this section contemplates a more intimate connection between this Association and other scientific bodies than exists at present. Naturally it would be supposed that the allusion is to medical bodies, or to bodies who deal with subjects cognate to medicine.

We cannot be blind to the fact that there is of late a tendency for each State to isolate itself professionally from every other, and to plan and work for the protection and for the advancement of the profession within its own borders rather than for the advancement of the profession at large. This movement, if carried to the ultimate, cannot but result badly, and it might be well to have this matter thoroughly discussed while it is yet in its incipiency. This state of things has no doubt been brought about by the fact that State societies practically keep aloof from each other, and we know as little of the medical societies of even our neighboring States as if they were separated by distances as great as China or Great Britain.

I submit to the judgment of the Association if it would not be to the benefit of the profession in general to inaugurate a system of representation at least in contiguous States, and request a similar action upon their part.

PROFESSIONAL SECRETS.

"A German practitioner has been sentenced to a fine of 75 lbs. ($375, N.) for unauthorized publication of secrets. He seems to have been exorbitant in his fees, but the offense with which he was charged was that of displaying in a public refreshment-room a bill of fees to a certain gentleman, which set forth that the attendance was on his wife for a sexual complaint. The laws in most continental countries distinctly recognize the obligation of medical men to keep the secrets of patients whom they attend professionally, and are as a rule duly enforced when necessary. * * * * * The question is based upon the great principle that the individuality of a private or hospital patient is secret. * * * * *"—*British Medical Journal, January* 16, 1886, p. 125.

In some countries (France and Germany), the revealing of a professional secret by a physician is criminal, and in many States (New York, Arkansas, California, Indiana, Michigan, Iowa, Missouri, Minnesota, Montana, Ohio and Wisconsin.—ATLANTA MEDICAL JOURNAL, January, '86, p. 702), they are by law made inviolable. Georgia is in this matter still in the rear rank, and it may be well for this Association to take into consideration the advisability of urging the passage of such a law.

The Correctional Tribunal of the Seine has just given judgment in an action brought against a medical man for violation of professional secrets. A discussion having arisen as to the cause of the death of M. Bastien Lepage, the famous painter, Dr. Watelot, who attended him in his last illness, wrote a letter to *Le Matin*, in which the malady in question was stated. Upon this the representatives of M. Bastien Lepage took proceedings.

The defendant pleaded that he meant no harm; but the court, while admitting that he was actuated by no unworthy motives, sentenced him to pay a fine of 100 francs, and the manager of the *Matin* to pay one of sixteen francs. The Tribunal laid down the principle that to constitute a misdemeanor it was not necessary that mischievous intentions should be proved. Even if a professional secret had been revealed with a laudable object the misdemeanor would none the less exist.

### SELLING POISONOUS DRUGS UNDER THE FORM OF SECRET REMEDIES.

The practice of selling poisonous secret remedies to the ignorant public is one carrying with it no little danger. In my own experience, one death has occurred during the past year, which could be attributed to no other cause; but as the purchase was made in another State nothing could be done in the matter.

One of the means of remedying this evil is by State legislation, but the true remedy lies in congressional action, which could at once make a general law applicable over the whole country.

### EXPERT TESTIMONY.

The status of professional men when called upon to testify as experts; the line of demarcation between material evidence and expert testimony; the amount of remuneration to be paid for the latter, and how it should be collected are questions which have already been before the "committee of legislation."

It is to be hoped that the committee will keep this matter constantly in view and be prepared to report thereon from time to time.

The following ruling of Judge C. C. Fuller, Mecosta county, Michigan: In the case of the People *vs.* Vaninmins, for the murder of John Crow, is of particular interest. In this connection a physician was called to testify on behalf of the defendant, and when asked for a professional opinion refused to answer until a fee for expert testimony was secured. The Judge said he had no authority to secure the fee; the bill must go before the Board of Supervisors. Witness said he had had enough experience with that body to trust them no further. The Judge said this question has never been decided in this State. "I can insist," he said, "on the witness answering, and if he then refuse I can fine him for contempt of court, and then let him fight it out with the Supreme Court, but after many years' study and observation I decide that a physician's knowledge is his stock in trade, his capital, and we have no more right to take provisions from a grocery without pay to feed the jury. The court rules that the witness is not compelled to testify." The doctor at once stepped off the stand. The defendant took exception to the ruling of the court. (From *Medical Age*, *Vol. IV.*, *No. 6*, *March 25*, *1886*, *p. 123.*)

I also append this clipping:

FEES OF THE COUNSEL.

WASHINGTON, April 5.—Solicitor-General John Goode appeared before the telephonic investigating committee this afternoon to tell what he knew of the circumstances leading up to the institution of the Memphis suit. . . . Mr. Lowery, who occupied an exceptional position in his capacity of electrical expert, was to receive a retainer of $1,000 and per diem of $70 when in New York taking testimony and $100 when called upon to leave that city, five hours being called a day.

PUBLICATION OF THE TRANSACTIONS.

A matter of profound importance to the Association, and one most difficult to handle, is the mode of publishing the transactions.

There are two forms in which the transactions can be published: 1st. As a separate volume, as was done before 1885, or 2nd, as a journal, as was done last year.

There are many advantages in publishing the transactions in separate volumes: 1st. They are unmixed with other matter. 2nd. The individuality of the doings of the Association is better preserved. 3d. The volumes, being bound—and well bound—are better preserved than is the pamphlet form of a journal. 4th. The risk of losing a number and thus spoiling the set is eleven-twelfths less than that of a monthly journal. 5th. Lengthy papers need not be divided. 6th. Convenience of reference.

The disadvantages incident to the annual volume are, 1st. The difficulty of getting together all the papers. 2d. The fact that one procrastinator may delay the publication of the volume for a long time; thus, all the papers read at the last meeting have not yet been published, simply because the authors elect to retain them in their hands for twelve months. 3d. Through this unavoidable delay much of the value of the papers is lost; the world moves too quickly now-a-days to admit of such delays. What is new and original and valuable to-day may in a year be utterly worthless in the light of later discoveries. 4th. There is no incentive to authors to write if they know their thought will remain bottled up for a year. 5th. The readers of our essays are only the members of the Association, 252 in number. It is not much of a stimulus to an author to know that his effort will reach the eye of but two or three hundred medical men. 6th. The sphere of usefulness of the Association is sadly curtailed—nay its very existence might be unknown outside its own membership. 7th. The Association has not got the money at its command to publish its transactions in the form of an annual volume, so that it has been necessary to solicit advertisements to aid in the publication. The Treasurer's report in 1884 shows the Association to have then been in debt $199.51. The report of 1885 gives no figures relating to the subject. 8th. The Association has to assume the risk of printing copies of the proceedings for delinquent members. 9th. It provides no means of inter-communication between the members.

THE JOURNAL SYSTEM.

The advantages of the journal are, 1st. Prompt publications of the proceedings—last year they appeared in May. 2d. Stimulating the authors of papers to promptness by publishing the papers in the order of their reception. 3d. Protecting the prompt writer by removing him from the retarding influence of the procrastinator. 4th. Giving to members at once such material as is on hand and the rest in the order of its reception. 5th. The Association takes no risk whatever, but makes a sure profit per *capitum* on its membership whether small or large. 6th. It furnishes a means of surely and certainly reaching the members whenever the occasion arises in the *interim* between the meetings of the Association. 7th. The proceedings reach a large number of non-members and show that this is a live Association, and thus we may hope that interest in the Association may increase, its membership be enlarged and its influence and power extended.

The disadvantages of the journal are, 1st. The flimsy binding. 2d. The increased risk of losing numbers. 3d. The division of lengthy articles into two or more parts. 4th. The chance that the Association may be accused of partisanship in the selection of a medium of publication. 5th. The interlarding of the proceedings of the Association with a quantity of foreign matter. 6th Inconvenience of reference.

The success of the *British Medical Journal*, the organ of the British Medical Association, may be cited as a fair reply to some of these disadvantages.

Reviewing the whole subject carefully, in my judgment the preponderance of advantages is greatly in favor of the journal form.

### SUGGESTIONS AS TO AWARDING THE PRINTING.

If the journal system is determined upon, I would suggest that the publication be awarded to the bidder offering to the Association the most favorable terms, and that in making a selection the Association or its committee shall be guided by the following considerations: 1st. Extent of circulation, that our proceedings may be read by the greatest number. 2nd. Lowest cost to the Association, that there may be an annual income accruing to the Association from its membership to defray incidental expenses that may arise. 3rd. The subscription price of the journal should be as near the price of the annual subscription to the Association as possible, that we may not give a premium to readers to keep out of the Association by furnishing our proceedings to non-members at a lower rate than they are furnished to our own membership.

Bids might be received and considered in two forms—either *en masse* for the whole number necessary to supply the wants of the Association, or at a rate *per capitum*, as at present. In either case the expenses of the postage, stationery and other printing (such as circulars, etc.) for the Association should be included.

We must not shut our eyes to the fact that upon this subject conflicting business interests will clash, and it therefore behooves the Association to approach this subject with a determination to do full justice to the Association and ignore all other issues.

In the past this Association suffered by dissensions caused by party strife. I pray you not to lose sight of the fact that this is a State Association, existing by the will of the profession of the State for the benefit of the whole. It is not the creature of any city, county or section, nor any college or journal, or printing-office, or clique of any kind, and I beg that you will frown down any effort upon the part of any officer or member to make this anything but a State Medical Association.

I consider it my duty to make these remarks because I appreciate the difficulty of making decisions without being swayed by inclinations, and I feared that through good nature or personal friendships the interests of the Association might be jeopardized.

### THE ASSOCIATION DORMANT.

For some reason to me unknown, the Association has not been increasing in numbers and in influence with the rapidity which its friends would desire and its importance deserves. The general profession do not manifest the same interest in it that they should. They show to it an apathy not at all in keeping with the relations borne to them by the Association.

It is not my desire to enlarge upon this subject, but simply to direct your attention in this channel.

It has been suggested to me that our plan of organization is at fault, and the remarkable success of the Medical Association of the State of Alabama has been instanced as an example for us to follow. Acting upon this suggestion, I wrote to Dr. Jerome Cochran, the senior censor of the State Association, and the moving spirit of the Alabama system, asking for information as to the organization of the Alabama Association.

I append extracts from Dr. Cochran's reply, which convey valuable and interesting information for which we cannot but feel under obligations to writer.

Should this Association consider it advisable to appoint a committee to investigate this subject, members might be found residing near the border of Alabama, who would undertake the task with but little personal inconvenience. Pending an investigation, I have no suggestions to make.

### EXTRACTS FROM LETTERS OF DR. COCHRAN ON THE ORGANIZATION OF THE MEDICAL ASSOCIATION OF THE STATE OF ALABAMA.

"The Medical Association of the State of Alabama has succeeded so remarkably for two reasons—

(1). In its organization and discipline it approximates a military standard and ignores all the ordinary clap-trap of freedom and equality. We have a Board of Censors who manage everything. We have a College of Counselors one hundred in number who pay the expenses and do the work of the Association. The county societies hold charters from the State Association, and are strictly subordinate to it, and their members are *ipso facto*, members also of the State organization. We do business in a business way. We pay our Secretary and our Treasurer, and the last gives bond in the sum of three thousand dollars for the faithful performance of his duty. We have a regular ritual for the revision of the rolls—the roll of the county societies—the roll of counselors—the roll of correspondents—the roll of officers. At their revisions every county society is called and must give an account of itself. If the account is not satisfactory it is referred to the Board of Censors for investigation, and if it proves incorrigible we take away its charter. Every counselor also is called and must be accounted for; if he has failed in payment of dues, or in attendance, without hesitation his name is struck from the roll and some one elected in his place. If he was old, revered, influential and had given years of service to the Association, still there would be no hesitation. His head would go—in a word we enforce our rules without respect of persons, and our order of business is such that nothing is overlooked or neglected. We are not liberal—we are strict, rigid—we are not generous—can't afford to be generous—we are simply just. As far as possible—I fear it cannot be done entirely—we banish all wire-pulling and office-seeking.

"(2). Our Association and our county societies have something to do—have a good deal of important work to do—and they do it. There is no bond like this to hold men together. We have entrusted to us by the State the administration of the law to regulate practice - and of the health laws of the State. We have an Examining Board and a Board of Health in every county—as well as a State Board of Examiners and of Health—all under the jurisdiction of the State Association. The County Boards examine only those who have diplomas, and reject a great many of these too. The State Board examines non graduates. Eaech one of our county societies, large or small, is allowed two delegates. The delegates and the counselors constitute the voting body of the Association, and a counselor must be a member of a county society. One main purpose we have in view is to maintain and magnify the usefulness of the county societies.

"Associations fail for various reasons: Greatest reason of all, they too often have no sufficient *raison d'etre*. Next most important reason, lack of discipline, which leads to a third reason, neglect. Men are lazy, doctors especially. They won't work."

Dr. Wm. Abram Love has been appointed Fraternal Delegate to the Medical Association of the State of Alabama, and from him we may expect a most interesting, valuable and suggestive report.

### DELAY OF AUTHORS IN FURNISHING COPIES OF ESSAYS.

The Committee on Publication have been sadly hampered and impeded in their work during the past year by the delay of authors to send in "copy." Some of the papers read by title at the last annual meeting were only sent to the committee last month. Amendment VII. to Constitution and By-Laws reads:

"All papers intended to be published in the transactions of this Association shall be placed in the possession of the Secretary within two weeks after adjournment."

The members will see the utter impossibility of successfully editing the transactions unless the amendment just quoted is strictly complied with. Thus it is some of the papers read in session of 1885 will appear in the transactions of 1886, to the great mortification of the Committee of Publication, who will receive all the censure which should justly fall upon the procrastinating authors.

I feel confident that it is only necessary to direct the attention of the members to this matter to have it remedied at once.

### DEFECTS IN THE BY-LAWS REGULATING THE PRESIDENT'S TERM OF OFFICE.

The By-Laws of this Association provide that the "President, Vice-Presidents and Censors shall enter upon the duties pertaining to their office at the commencement of the annual meeting succeeding their election."

I desire to express the opinion that in the case of the President the operation of this By-Law works to the detriment of the Association, in that it takes from the officer who is to preside over your annual meeting the power to speak authoritatively to the members until the assembling of the body; therefore the real work of getting up a good meeting, if any is done, must be done by the retiring President, and done for the benefit of his successor.

The only advantage that can possibly come from the present method is that it gives the President a year to prepare an address; but this could just as well be made the duty of the retiring President, and being a personal matter would be done by him just as well upon his retirement as upon his inauguration.

Of many methods which suggest themselves for the correction of this evil I will mention but two:

(1.) Let the President enter upon the duties of his office immediately upon the adjournment of the meeting at which he is elected, and let the retiring President deliver the annual address, which shall include an account of his acts during the year preceding and suggestions for the improved working of the Association.

(2.) No matter how arranged, let the retiring President, when introducing his successor, give to the Association an account of his stewardship, of his official acts during his year of office, with such suggestions as his experience would dictate as being useful to increase the efficient working and influence of the Association.

No officer would venture to come before this Association and acknowledge that its trust had been misplaced, and that during his year of office the Association had been dormant; that he during the year passed had done nothing to stimulate professional interest in the Association; that he had not raised his hand to add to the influence of the Association; that no effort had been made by him to enlarge the attendance at our meetings nor any act of his done to augment the scientific value of our gatherings, nor to increase our membership.

By the present arrangement the Association absolutely deprives itself of any advantage it might derive from suggestions which could be made by the retiring President touching the working of the Association because he virtually has none upon which to found an opinion.

As this question is only one of By-Law it can be amended by a two-thirds vote of this meeting if the Association should desire to do so.

Of course the change will shorten the official term of the present incumbent, but I am not only willing but anxious that it should be done, because I believe it will redound to the benefit of the Association by throwing in a great measure upon the shoulders of the presiding officer the responsibility for the success of the meeting.

### PATENTS.

A somewhat extended inquiry into the opinions held by progressive physicians in various parts of the country has convinced me that there is an increasing dissatisfaction with that portion of the "Code of Medical Ethics" regulating the holding of patents by physicians, which mixes up patents for surgical instruments, patents for medicines and secret nostrums in inextricable confusion. The language of the Code is as follows:

"SEC. IV. Equally derogatory to professional character is it for a physician to hold a patent for any surgical instrument or medicine, or to dispense a secret *nostrum*, whether it be the composition of exclusive property of himself or of others; for if such nostrum be of real efficacy, any concealment regarding it is inconsistent with beneficence and professional liberality; and if mystery alone give it value and importance, such craft implies either disgraceful ignorance or fraudulent avarice. It is also reprehensible for physicians to give certificates attesting the efficacy of patent or secret medicines, or in any way to promote the use of them."

### INSTRUMENTS.

Some of the objections urged to including surgical instruments in this prohibitory section are: 1st. That while physicians may not patent instruments in the United States, it is not unethical to do so in other countries. 2nd. Foreign physicians and inventors generally, as for example surgical instrument-makers, do patent surgical instruments, both in this country and abroad, and these instruments are freely purchased and used by regular physicians. 3rd. That it is impossible under the existing arrangement to insure that an instrument sold is just what it purports to be, each maker modifying the instrument to suit his own notion until the design of the inventor is entirely lost, although *he* will still be held responsible for all failures. 4th. That by no possibility can the originality of an invention be determined, so that pirating is common, and the same instrument is often claimed by numerous inventors. Other objections have been urged, but these are sufficient to show the line of argument.

### PROPRIETARY MEDICINES.

Many regular physicians now prescribe proprietary medicines which, while not professedly secret remedies, are exceedingly close to it, and much unfavorable comment has been thus occasioned. Certainly distinction is needed between a "patented medicine," a "proprietary medicine" and a "secret *nostrum*."

### PUFFS IN SECULAR PAPERS—CERTIFICATES GIVEN TO NOSTRUMS.

At the session of 1883 (see page 24 proceedings) the Association spoke in unmistakable terms of disapproval of the practice of giving certificates to patent or secret medicines and other similar practices in the following language:

"The Board of Censors consider it to be their duty to call the attention of the Association to the habit, which prevails to a great extent among members of the profession, of permitting their names to appear as indorsing patent or proprietary medicines, remedies, diets, mineral waters, etc., in which indorsement their official designation appears.

"Likewise would the board bring to the consideration of the Association the frequent appearance of paragraphs in secular papers, in which allusion is made, either by name or by unmistakable allusion, to physicians in connection with cases or other medical matters. The appearance of these indorsements and paragraphs is suggestive of connivance, by permission, if not direct personal influence, of the physician himself, in procuring its publication.

"Such conduct is certainly contrary to the spirit of medical ethics and the Board of Censors desire that the Association hereby express its condemnation of such practices."

There is little use in endeavoring to correct the evil in this State when from other parts of the country comes the evidence that prominent members of the profession are constantly violating the spirit and letter of the ethics in this particular.

It might be well that our delegates to the American Medical Association should be instructed to draw attention to this subject, that the Association may condemn such conduct, no matter what may be the prominence or influence of the offender, or else expunge that portion of the ethics from the Code as being a dead letter.

The following is the language of the Code of Ethics upon this subject:

"SEC. 3. It is derogatory to the dignity of the profession to resort to public advertisements, or private cards, or handbills inviting the attention of individuals affected with particular diseases, publicly offering advice and medicine to the poor, gratis, or promising radical cures, or to publish cases and operations in the daily prints or suffer such publications to be made; to invite laymen to be present at operations, to boast of cures and remedies, to adduce certificates of skill and success, or to perform any other similar acts. These are the ordinary practices of empirics, and are highly reprehensible in a regular physician."

### A PRESIDENTIAL PRIZE ESSAY AND ENDOWMENT FUND.

It goes without saying that it is the duty of those who are honored with the most prominent position which the medical profession of the State can bestow to do, each in his turn, something to advance the position which the Medical Association of Georgia occupies in the scientific world.

It is not enough that he to whom you entrust the guidance of the affairs of your Association should content himself with obeying the letter of your law and nothing more, that he should rest satisfied with reading an inaugural address and presiding over your annual deliberations.

Your Constitution says that "he shall in the intervals between the annual sessions direct and control the general policy and business of the Association." Clearly, then, it is the special duty of your presiding officer to have the best interests of the Association constantly before his mind, and to make to the body when annually assembled such suggestions as will in his opinion tend to advance the Medical Association of Georgia towards professional and scientific eminence.

One of the advantages to be derived from a frequent change of officers is that each year will bring to the Association some new idea, some new plan, which will permanently increase the professional influence of the Association and enlarge the sphere of usefulness.

No one will deny that original observations are the foundation of progress in medicine, and it follows that if this Association would be true to itself, and would study its own interests and that of the profession of the State of Georgia, it would make a persistent and determined effort to foster and encourage original research. This means money; for there are but two ways occurring to me by which it can be done: 1st. By grants from the Association, as is now done by the British Medical Association; and 2nd, by a permanent system of prizes of sufficient value to stimulate research to be offered for original essays. In the present financial condition of the Association, the first proposal is absolutely impracticable, but the second does not seem so difficult. I beg, therefore, to suggest to the Association the following plan:

1st. Let there be organized a system of annual prizes for original research and investigation, to be known as the President's prize, to be awarded under such rules and regulations as the Association may determine.

2d. Let it be understood that it is the sense of the Association that every President shall contribute one hundred dollars to this fund upon his inauguration.

By the adoption of this proposal the Association would be in the possession of an invested fund, constantly increasing in size, until it would be of such value as to enable the Association to amply reward an investigator for the labor and expense necessarily attendant upon original researches; to such a prize the Association might also add a life membership in case of the successful essayist being a pay member or subsequently becoming one.

I do not make this proposition for the purpose of affecting others and exempting myself from its operation; if the Association adopts this proposal, the Committee on Prize Essays is hereby authorized to add to the Prize Essay and Endowment Fund, as my contribution, the sum of one hundred dollars, which I will pay on their demand.

I know I will be met by the stereotyped objection that many good men are too poor to afford this. But by way of rejoinder, I would say that if one will assume honors he should be prepared to pay for them. If one wears a good coat he must pay for it; if one moves in respectable society he must pay for it—he must pay for his education, he must pay for his diploma, he must pay for everything.

Every President of this Association has twelve months' notice of his election, and he can stop his wines or tobacco or other luxuries to the amount of two dollars a week, and give the sum total to the President's Prize Essay Fund; and any man who appreciates the honor conferred upon him so little as not to be willing to make this sacrifice is, in my estimation, unworthy of the position.

In this very city there will assemble next month an association which is purely social in its character, yet in that body by mere force of custom the presiding officer feels compelled to annually offer one or more prizes for the exhibition of such characteristics or qualities as are considered of value to the society. Can it be possible that the President of the Medical Association of Georgia, the most important scientific body in the State, will object to doing that which is freely done by the officers of a social organization?

Is the highest scientific position in the State of Georgia of so little importance in the estimation of the members of this Association that each is not willing in his turn to make so trifling a sacrifice in return for the honor conferred upon him?

I trust the Association will express a favorable opinion of this suggestion, for there is much that we can do with a small invested fund. "All things come to him who can wait."

